# Comparative evaluation of compressive strength among different glass ionomer cements

**DOI:** 10.6026/973206300214625

**Published:** 2025-12-15

**Authors:** Puja Chavan, Yash Rajendra Shaha, Suyash Pratap Singh, Supriya Makam, Ashwini Komalan, Arti Gachake, Mrunal Dave, Pratik Surana

**Affiliations:** 1 Consultant Endodontist, The Dental Corner, Navi Mumbai, Maharashtra, India; 2 Department of Conservative Dentistry and Endodontics, Tatyasaheb Kore Dental College and Research Centre, New Pargaon, Maharashtra, India; 3 Department of Conservative Dentistry and Endodontics, Institute of Dental Sciences, Bareilly, Uttar Pradesh, India; 4 Department of Conservative Dentistry and Endodontics, Rajarajeswari Dental College and Hospital, Bangalore, Karnataka, India; 5 Department of Prosthodontics, Rural Dental College, PIMS, Loni, Ahmednagar, Maharashtra, India; 6 Department of Prosthodontics and Crown and Bridge, Bharati Vidyapeeth (Deemed to be University) Dental College and Hospital, Pune, Maharashtra, India; 7 Department of Aesthetic Dentistry, Bethlehem Smile Design, Bethlehem, Pennsylvania, USA; 8 Department of Pedodontics and Preventive Dentistry, Maitri College of Dentistry and Research Centre, Durg, Chhattisgarh, India

**Keywords:** Glass ionomer cement, amalgomer CR, zirconomer, compressive strength

## Abstract

The relatively low compressive strength of conventional glass ionomer cements limits their use in stress-bearing areas, necessitating evaluation of newer reinforced variant. Therefore, it is of interest to evaluate compressive strength of different types of glass ionomer cements (GIC): conventional GIC (Group A), Zirconomer (Group B) and Amalgomer CR (Group C). Each group consisted of 12 specimens formed in plastic straws with 4mm 6mm dimension. Compressive strength was assessed using a universal testing machine at a crosshead speed of 1 mm/min. Result showed that Zirconomer exhibited the highest compressive strength, indicating its superior performance compared to the other materials.

## Background:

Dental caries presents a significant community health concern worldwide, particularly within children. Caries can initiate early in life, progress rapidly in high-risk individuals and often goes untreated. When dental caries occurs, restoring the affected tooth turn out to be essential [1, 2]. Glass ionomer cement is the utmost used material for restoring primary teeth. Its unique properties, such as fluoride release, a coefficient of thermal expansion similar to that of natural tooth structure, chemical bonding to enamel and dentin, as well as excellent biocompatibility and aesthetics, make it a valuable tool in restorative dentistry. However, GICs are prone to fracture and have low wear resistance, which limits their applicability in intense areas, such as proximal restorations, rendering them less suitable for certain clinical situations [2, 3]. In 1977, amalgam alloy powder was integrated into glass ionomer cements to improve strength and radiopacity, but metal-reinforced cements have not become popular for tooth-colored restorations due to inadequate interfacial bonding, limiting their durability compared to metal-free versions [4]. In the late 1980s, the addition of polymerizable hydrophilic resins to conventional glass ionomers led to resin-modified formulations with better mechanical properties, though challenges like polymerization shrinkage and low wear resistance persist. Recently, Amalgomer CR, a novel ceramic-infused GIC, has been presented to the dental market and is being promoted for its enhanced performance in restorative applications [5, 6]. Zirconia-reinforced glass ionomer cement is a modern variant that incorporates zirconia, a extremely resilient ceramic. This material consists of zirconium oxide (powder) combined with polyacrylic acid and deionized water (liquid). The addition of zirconia fillers enhances the structural integrity, while the homogenization of glass particles contributes to superior mechanical properties and durability [7, 8]. The compressive strength of materials plays a crucial role in determining the durability and effectiveness of restorative treatments [3, 9]. With an increasing range of Glass Ionomer Cements on the market, it's essential to systematically assess and compare their mechanical properties. While numerous studies have examined these materials separately, there is still a lack of in vitro comparisons. Therefore, it is of interest to evaluate the compressive strength of three types of GICs: conventional GIC, Zirconomer and Amalgomer CR.

## Materials and Methods:

This study focused on evaluating and comparing the compressive strength of three types of glass ionomer cements: conventional GIC, Zirconomer and Amalgomer CR. We prepared 36 cylindrical specimens using plastic straws that measured 4mm in diameter and 6mm in height as molds. To prevent the materials from sticking, we applied petroleum jelly to the inner surfaces of the straws. Each type of GIC was mixed following the manufacturer's guidelines to ensure optimal properties. The prepared material was carefully placed into the molds arranged on a glass slide. To create a smooth and even surface, we covered the filled molds with a mylar strip and pressed them with another glass slide. This approach guaranteed consistent thickness and surface quality for each specimen. Each GIC type had 12 specimens, leading to three groups: conventional GIC (Group A), Zirconomer (Group B) and Amalgomer CR (Group C), as summarized in Table 1 (see PDF). The samples were removed from the molds after one hour and any excess material was carefully ground. Prior to assessment, the samples were stored in distilled water at room temperature for 24 hours. Compressive strength testing was done using a universal testing machine at a crosshead speed of 1 mm/min along the longitudinal axis of each cylindrical specimen. The maximum force at failure was recorded and compressive strength (in MPa) was calculated using the formula is C = 4p/Πd2, where P = maximum force (N), d = average specimen diameter (mm) and Π =3.141 is the average specimen diameter (mm). Data analysis was performed using one-way ANOVA followed by Tukey's post hoc test at a significance level of p < 0.05.

## Results:

The mean compressive strength values observed in this study indicated a statistically significant difference among the groups. Group B, which consisted of Zirconomer, exhibited the highest mean compressive strength at 159.11 ± 4.81 MPa. In contrast, Group A, containing conventional GIC, displayed a much lower mean compressive strength of 100.23 ± 3.22 MPa. Group C, represented by Amalgomer CR, had a mean compressive strength of 152.24 ± 3.66 MPa. Statistical analysis confirmed that all inter-group comparisons between the mean compressive strengths were significant, demonstrating that Group B (Zirconomer) outperformed both Group A (conventional GIC) and Group C (Amalgomer CR). Notably, the comparison between Group B and Group C, while suggesting a higher mean for Zirconomer, did not reach statistical significance (P > 0.05). This indicates that while Zirconomer has superior performance compared to conventional GIC, the difference in strength between Zirconomer and Amalgomer CR was not enough to be deemed statistically significant (Table 2 - see PDF).

## Discussion:

Dental Caries frequently result in the necessity for restorative procedures since they damage the tooth's structural integrity. Restorative solutions must endure the constant chewing forces within the mouth for long durations, requiring them to be both robust and fracture-resistant. When assessing dental materials, important factors include their ease of use, mechanical characteristics and adaptability. A crucial mechanical aspect is compressive strength, as these materials often take the place of substantial portions of tooth structure and must be sufficiently strong to withstand the pressures exerted during mastication [10, 11]. The objective of this research was to compare the compressive strength of three types of restorative materials. The results revealed statistically significant differences among the groups, with Group B (Zirconomer) demonstrating superior compressive strength compared to Groups A and C. These results underscore how the material composition can greatly affect the mechanical performance of restorative materials. Zirconomer Improved is a cutting-edge restorative option that fuses ceramic and zirconia-reinforced glass-ionomer cement. It is designed to be a reliable and durable self-adhesive solution in tooth color, ideal for posterior bulk-fill restorations. The incorporation of nano-sized zirconia fillers enhances both the handling properties and aesthetic appeal of this innovative material [12]. In our research, the Zirconomer group showed the strongest compressive strength. Likewise, a study conducted by Bhatia HP and their team analyzed traditional GIC, silver-reinforced GIC and zirconomer, concluding that zirconia-reinforced glass-ionomer cement had the highest compressive strength of all the materials examined [13]. In a similar study, Nagar and co researcher compared three different esthetic restorative materials: Zirconomer, resin-modified GIC and conventional GIC. They found that Zirconomer exhibited the highest compressive strength among the options. As a result, Zirconomer may be the preferred choice for aesthetic restorative procedures [14].

This result can be accredited to the homogeneous integration of small sized zirconia particles into the glass component, which enhances the material's strength, durability and ability to withstand occlusal loads. Additionally, zirconia exhibits transformational toughening, a property that helps prevent the growth of cracks, thereby contributing to its unique mechanical characteristics [15, 16]. In our study, the amalgomer CR group also showed results comparable to those of zirconomer. Wang Y & Darvell BW suggested that it may be attributed to the filler content, primarily quartz in GIC, which is reinforced by ceramic particles in Amalgomer CR. As per manufacture, these ceramic fillers can partially react with the matrix, potentially producing some bonding that enhances the overall strength of the restoration [17]. Conventional glass ionomer cement exhibited the lowest compressive strength due to its formulation and the inherent properties of the materials used. Factors such as the composition, the setting reaction and the bonding mechanisms can contribute to lower strength compared to other materials. Additionally, it's handling characteristics and moisture sensitivity during the curing process may also play a role in its overall performance [18, 19]. Limitations of our study include a focus on a single specimen dimension (6 x 4 mm); thus, the effects of varying specimen dimensions on compressive strength warrant further investigation. Additionally, while this study examined compressive strength, it did not address other mechanical properties of the cement, such as tensile strength, flexural strength, or wear resistance, which should be explored in future research. Other limitations include a small sample size, which may affect the generalizability of the findings and the lack of long-term evaluation, as the durability and performance of the materials over time were not assessed. Furthermore, the study was conducted under controlled laboratory conditions, which may not accurately reflect clinical scenarios.

## Conclusion:

We show the quality of each material used, emphasizing their clinical significance. Among the tested groups, Zirconomer stood out with the highest compressive strength, trailed by Amalgomer CR, which secured the second position. On the other hand, conventional glass ionomer cement demonstrated the weakest compressive strength.
